# A Lapse

**Published:** 1871-04

**Authors:** 


### A LAPSE.

In an article in the *Dental Times*, Prof. Buckingham, tells us how to reduce gold and silver scraps to plate. When the silver is reduced to a chloride, he says: "The chloride should be covered with fresh water, and add a small portion of sulphuric acid; then put a few small pieces of zinc in the vessel, and let it stand for a day. The chlorine will leave the silver and combine with the zinc."

The closing sentence contains the lapse. The zinc decomposes the water, taking oxygen, and liberating hydrogen. The hydrogen takes the chlorine from the silver. As the oxyd of zinc is formed, it unites with the sulphuric acid. The liquid contains sulphate of zinc, not chloride; and the escaping gas is hydrochloric acid, not hydrogen. And Prof. B. knew all this as well as anybody, and he should have written more carefully, in writing for the young, as he proposed to do. W.

